# Implications of Oncoplastic Breast Surgery on Radiation Boost Delivery in Localized Breast Cancer

**DOI:** 10.7759/cureus.20003

**Published:** 2021-11-29

**Authors:** Adam Gladwish, Giulio Didiodato, Jessica Conway, Christiaan Stevens, Matthew Follwell, Tiffany Tam, Jesse Mclean, Renee Hanrahan

**Affiliations:** 1 Oncology, Royal Victoria Hospital, Barrie, CAN; 2 Internal Medicine, Royal Victoria Hospital, Barrie, CAN; 3 Radiation Oncology, Royal Victoria Hospital, Barrie, CAN; 4 Medical Education, Royal Victoria Hospital, Barrie, CAN; 5 Surgery, Royal Victoria Hospital, Barrie, CAN

**Keywords:** breast cancer, boost radiotherapy, breast-conserving surgery, oncoplastic surgery, breast radiotherapy

## Abstract

Background

Oncoplastic partial mastectomy (OPM) is a technique utilized to improve aesthetic and survivorship outcomes in patients with localized breast cancer. This technique leads to breast tissue rearrangement, which can have an impact on target definition for boost radiotherapy (BRT). The aim of this study was to determine if the choice of surgical technique independently affected the decision to deliver a radiation boost.

Materials and methods

This was a retrospective study of patients treated between January 2017 and December 2018. We selected consecutive patients based on surgical procedure: 50 undergoing standard breast-conserving surgery and 50 having had an OPM. The primary outcome was average treatment effect (ATE) of surgery type on reception of BRT. Secondary outcomes included ATE of surgery type on the time to reception of radiotherapy and incidence of ipsilateral breast tumor recurrence (IBTR). The ratio of boost clinical target volume (CTV) to pathologic tumor size was also compared between the two groups. Treatment effects regression adjustment and inverse-probability weighted analysis was used to estimate ATEs for both primary and secondary outcomes.

Results

For the entire cohort, the median age was 64 years (range: 37-88 years). The median tumor size was 1.5 cm (range: 0.1-6.5 cm). The majority of patients were with ≤ stage IIA (78%), invasive ductal subtype (80%), negative lymphovascular space invasion (78%), negative margin (90%), and positive ER/PR (estrogen receptor/progesterone receptor) (69%). Overall, surgical technique was not associated with differences in the proportion of patients receiving BRT (ATE: 6.0% [95% CI: -4.5 to 16.0]). There were no differences in delays to radiation treatment between the two groups (ATE: 32.8 days [95% CI: -22.1 to 87.7]). With a median follow-up time of 419 days (range: 30-793 days), there were only five recurrences, with one case of IBTR in each group. There was no difference in the ratio of CTV volume to tumor size between the two groups (p=0.38).

Conclusions

OPM did not affect the decision to offer localized BRT following standard whole breast radiotherapy or significantly affect treatment times or radiation volumes. The decision to offer OPM should include a multi-disciplinary approach.

## Introduction

Breast cancer is the most commonly diagnosed malignancy in women in Ontario, representing more than 10,000 new cases in 2017 alone [1]. The management for the majority of these patients includes lumpectomy and adjuvant whole breast radiotherapy, which has been widely accepted as the standard of care [2]. An additional boost of radiation localized to the tumor cavity in invasive disease has been shown to reduce ipsilateral breast tumor recurrences (IBTR), particularly in patients of younger age or with high-grade tumors [3,4]. Oncoplastic partial mastectomy (OPM) procedures have become increasingly prevalent as a means to improve the quality-of-life outcome for patients [5-7]. Oncologic outcomes such as local recurrence and survival have been shown to be similar to standard lumpectomy techniques [8,9]. It has been theorized that the technical aspects of oncoplastic surgery may hinder the ability of radiation oncologists to accurately identify the post-operative cavity, which is required to accurately deliver local boost treatments [10].

The impact of OPM on the administration of adjuvant boost radiotherapy (BRT) has not been studied. Several studies have shown favorable results of oncoplastic surgery in terms of local control rates, but they were inclusive of patients with lower baseline risks of recurrence for whom BRT would not typically be indicated [8,11]. Larger retrospective reviews have a paucity of radiation planning details [12]. The aim of this retrospective study was to evaluate the impact of oncoplastic breast-conserving surgery (BCS) on the likelihood of receiving BRT. We hypothesized that undergoing OPM would be an independent negative predictor of BRT.

This article was previously published as a preprint (https://www.researchsquare.com/article/rs-33599/v1).

## Materials and methods

Study population

**Figure 1:**
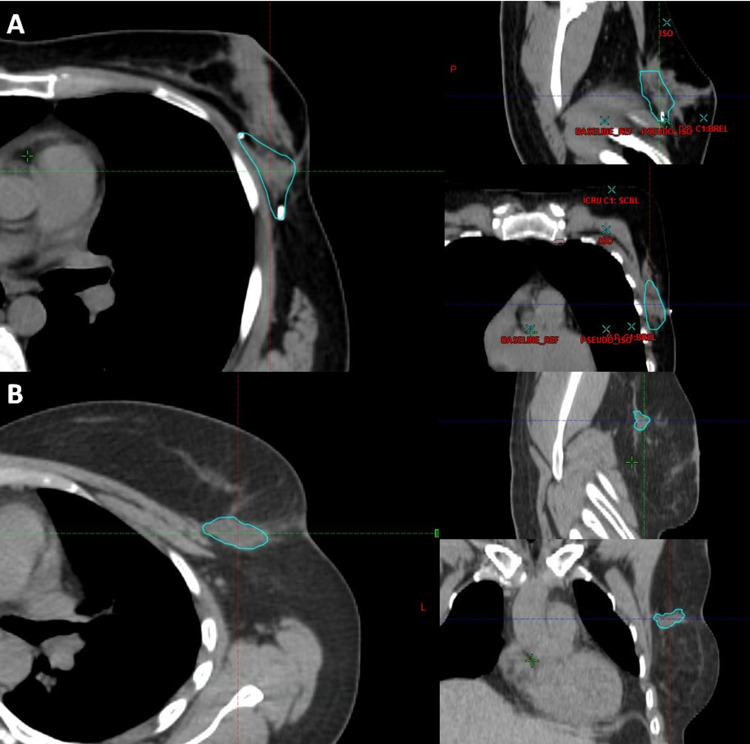
Representative image of CTV for (A) OPM and (B) BCS. CTV, clinical target volume; OPM, oncoplastic partial mastectomy; BCS, breast-conserving surgery

A retrospective medical record review was approved by the Royal Victoria Hospital Research Ethics Board. We estimated that we needed to review at least 100 medical records in a 1:1 ratio of OPM:conventional BCS to detect an average treatment effect (ATE) of ≥ 15% difference on proportion of patients receiving BRT between the two groups with a power of 80% and an alpha of 0.05%. Patients with stage I-III invasive carcinoma of the breast treated with OPM or BCS from January 2017 to December 2018 were identified. Charts were selected in a sequential fashion until 100 records meeting study criteria were filled. Exclusion criteria included patients with mastectomies, male patients, patients with non-invasive disease (i.e., ductal carcinoma in situ), patients receiving accelerated partial breast radiation, and those under the age of 18. All patients underwent partial mastectomy in the standard form of full-thickness resection from anterior to posterior planes, including resection of the pectoralis fascia. Surgical resection beds were marked with a minimum of six clips on the posterior wall and parenchymal edges. OPM included closures that were completed utilizing a variety of oncoplastic techniques including both advancement and rotational flap closures. All patients received our institutional standard adjuvant whole breast radiation with 4,256 cGy in 16 fractions given once daily, five days per week. When BRT was prescribed, patients received 1,000 cGy in four subsequent fractions, delivered to the clinical target volume (CTV), delineated by a radiation oncologist, intended to include radiographic seroma and all relevant surgical clips, particularly in the case of OPM, with an additional isotropic margin of 1 cm (planning target volume). Figure 1 illustrates CTV in a representative case of OPM and BCS.

Data collection

Patient demographics (age at diagnosis, date of diagnosis, disease histology, grade, tumor size, lymphovascular space invasion, hormone receptor status, laterality, and stage), treatment characteristics (chemotherapy, margin status, surgery type [OPM or BCS], date of surgery, radiation boost, date of last clinical follow-up), and radiation data (radiation date, radiation dose, CTV volume) were extracted.

Statistical analysis

Baseline patient, treatment, and tumor characteristics were compared using the t-test test or chi-square test depending on the variable type.

The primary outcome of this study was to compare the proportion of patients who received BRT administration between the two surgical groups. In order to facilitate analysis of the primary outcome, the breast boost indication needed to be standardized across both groups. For the purposes of this study, BRT indications were defined as one of following conditions: age ≤ 50 years; age 51-60 years with ≥1 high-risk feature; age > 60 years with ≥ 2 high-risk features. High-risk features included grade 3 histology, presence of lymphovascular space invasion, positive margins, and negative estrogen receptor (ER) status. Agreement between “BRT indicated” status and reception of BRT was estimated using a kappa statistic. The primary ATE was estimated using treatment effects inverse-probability weighted logistic regression adjustment of BRT. The outcome variable was binary as to whether a patient received BRT (yes/no). The type of surgery was also modelled as a binary variable and was included as the intervention variable in the regression model. Age and tumor size were included as continuous variables in the model. The type of surgery included in the model was weighted on age and tumor size to minimize surgical type selection bias, and reception of BRT was conditioned on age and reception of chemotherapy. Tumor grade, lymphovascular space invasion, positive margins, and ER status were found to be nearly perfectly correlated with reception of BRT and so were not included in the model.

Secondary outcomes included comparative analyses of time to BRT, incident case of IBTR, and CTV volume relative to pathologic tumor size between the two surgical groups. The latter metric was included in order to try and characterize if there were systematic influences on boost volume as a result of the type of surgery (i.e., were oncologists consciously or subconsciously accounting for surgical procedure via radiation volumes). Follow-up was defined from the date of surgery to last clinical follow-up visit. Time to radiotherapy was defined from the date of surgery.

The secondary ATE on time to reception of adjuvant breast radiotherapy was estimated using treatment effects inverse-probability weighted survival regression adjustment. The type of surgery was included as the intervention variable. Type of surgery was conditioned on the same variables as previous. The time to radiotherapy was conditioned on age (continuous), reception of chemotherapy (binary), and CTV volume (continuous). Two-sample Wilcoxon rank-sum test was used for the comparative analysis of the ratio of CTV volume to maximal pathologic dimension between the two surgical groups.

STATA/MP 15.0 for Mac (StataCorp LLC, College Station, TX) was used for all analyses.

## Results

Median follow-up for the entire cohort was 419 days (IQR: 30-793 days). One patient in the standard BCS group had synchronous bilateral breast cancer, and therefore 51 tumors were evaluated for these 50 patients. Patients undergoing OPM were generally younger (median age 60 vs. 69 years; p = 0.003) and had higher stage tumors (stage > 1A 44% vs. 61%; p = 0.049). OPM patients were also more likely to have had positive surgical margins (14% vs 6%; p=0.047) and have received intravenous chemotherapy (84% vs. 52%; p=0.001) and radiation boost (40% vs. 22%; p=0.025). All patient, tumor, and radiotherapy characteristics are detailed in Table 1.

**Table 1:** Demographics BCS, breast-conserving surgery; OPM, oncoplastic partial mastectomy; LVSI, lymphovascular space invasion; ER, estrogen receptor; PR, progesterone receptor; HER2, human epidermal growth factor receptor 2

Variable	Standard BCS	OPM	p-Value
Age (years)			
Median (IQR)	69 (60–71)	60 (52–67)	0.003
Site			
Left	27 (53%)	20 (40%)	0.229
Right	24 (47%)	30 (60%)	
Grade			
G1	19 (37%)	17 (33%)	0.091
G2	17 (33%)	25 (50%)	
G3	15 (30%)	8 (16%)	
Histology			
Invasive ductal	43 (84%)	37 (74%)	0.374
Invasive lobular	3 (6%)	8 (16%)	
Other	5 (10%)	5 (10%)	
Size (greatest dimension, cm)			
Mean (SD)	1.47 (0.85)	1.76 (1.09)	0.138
LVSI			
Yes	5 (10%)	7 (14%)	0.726
No	41 (80%)	38 (76%)	
Indeterminate	5 (10%)	5 (10%)	
Margin			
Positive	3 (6%)	7 (14%)	0.047
Negative	44 (86%)	47 (94%)	
Stage			
IA	31 (61%)	22 (44%)	0.049
IB	8 (16%)	16 (32%)	
IIA	7 (14%)	3 (6%)	
IIB	4 (7%)	5 (10%)	
IIIA	1 (2%)	3 (6%)	
IIIB	0	1 (2%)	
Receptor status			
ER/PR +ve, HER2-	38 (75%)	35 (70%)	0.807
ER+ve, PR/HER2-	3 (6%)	7 (14%)	
ER/PR –ve, HER2+	1 (2%)	2 (4%)	
Triple positive	5 (10%)	3 (6%)	
Triple negative	4 (7%)	3 (6%)	
Chemotherapy			
Yes	26 (52%)	42 (84%)	0.001
No	24 (48%)	8 (16%)	
Radiation boost			
Yes	11 (22%)	20 (40%)	0.025
No	40 (78%)	30 (60%)	
Boost volume (cc)			
Mean (SD)	25.62 (38.31)	26.34 (36.25)	0.987

There was 98% agreement between BRT indication criteria and reception of BRT (kappa=0.95) (Table 2). The primary ATE was 6.0% (95% CI: -4.5 to 16.0), and fewer patients received BRT in the OPM group. The secondary ATE for time to reception of radiotherapy was 32.8 days (95% CI: -22.1 to 87.7) longer in the OPM group, with a mean of 123.2 days (95% CI: 88.5 to 157.9) in the BCS group. The primary and secondary ATEs had confidence intervals spanning 0, indicating no statistically significant effect. All primary and secondary ATEs are given in Table 2. With only five recurrences (two local and three distant) among the entire group and only two ipsilateral breast tumor recurrences (one from each group), there were not enough events to assign meaningful oncologic outcome statistics.

**Table 2:** Primary and secondary ATEs ATE, average treatment effect

Outcome	Control mean (95% CI)	ATE (95% CI)
Primary: proportion of patients receiving BRT	0.32 (0.22 to 0.43)	-0.06 (-0.16 to 0.045)
Secondary: time (days) from surgery to receiving BRT	123.2 (88.5 to 157.9)	32.8 (-22.1 to 87.7)

**Figure 2:**
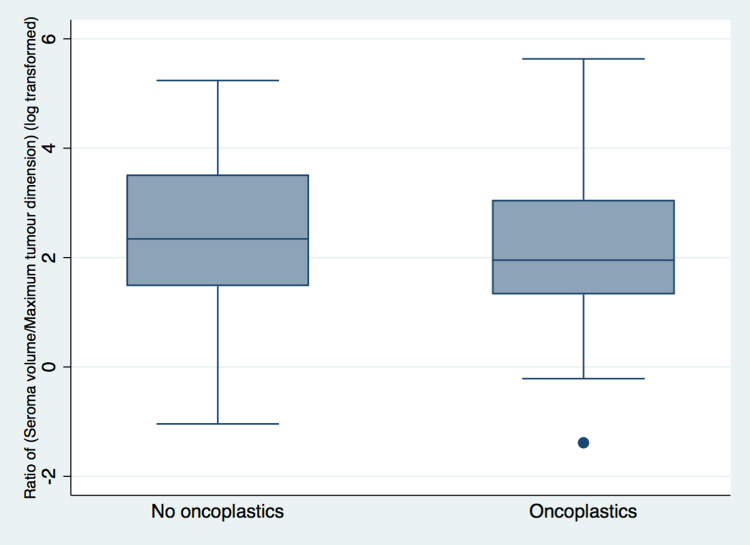
Box plot comparing OPM (oncoplastics) with BCS (no oncoplastics) using ratio of CTV volume to maximum pathologic dimension. OPM, oncoplastic partial mastectomy; BCS, breast-conserving surgery; CTV, clinical target volume

There were no differences in the ratio of CTV volume to tumor size between the two groups (p=0.38), suggesting that boost volumes were not systematically altered (larger or smaller) in patients who underwent OPM (Figure 2).

## Discussion

The favorable oncologic and aesthetic outcomes associated with OPM have led to an increase in uptake of these procedures [7,13]. There have been well-described concerns in the literature about radiation boost delivery and accurate localization for these patients [14-16]. This study aimed to identify if there was a propensity to omit BRT as a result of surgical technique, presumably for concern regarding localization. Not surprisingly, given the typical patient selection for OPM, we did find that there were differences between the groups in terms of patient age, stage, margin status, and chemotherapy administration, which likely resulted in significantly more patients within the OPM group receiving BRT. However, when conditioned on BRT indication criteria, there were no significant differences found in the proportion of patients receiving BRT. This suggests that the recommendation of BRT was predicated on tumor characteristics rather than surgical technique. We also found no significant difference in delays to adjuvant radiotherapy. Any potential differences in incidence of IBTR could not be estimated due to the relatively short follow-up times and very few IBTR events, which were limitations also noted in previous studies [8,11,14,15,17,18]. Finally, we evaluated the relationship between the ratio of the CTV volume to tumor size and reception of BRT. The rationale was to assess whether a radiation oncologist might alter the BRT volumes (either knowingly or subconsciously) in the context of OPM and the known associated architectural distortion. Interestingly, there were no significant differences seen in this measure, which could suggest radiation oncologist utilized supplementary means (in addition to CT simulation and operative clipping) in volume definition. These could reasonably include any pre-operative imaging (mammography, CT staging, or MRI), operative notes, and involvement of the surgeon’s input directly at the time of contour delineation.

To our knowledge, this is the first study reporting on whether surgical technique influences the decision to proceed with radiation boost delivery in adjuvant breast radiotherapy. A systematic review by Schaverien et al. demonstrated that the majority of studies reporting on oncologic outcomes of OPM did not provide sufficient radiotherapy details regarding the application of BRT to assess whether OPM impacted delivery of BRT [12]. This was despite the propensity of OPM to be performed in younger patients with more advanced tumors, a characteristic shared in our study as well. Similarly, a recent retrospective study of 965 patients by Borm et al. showed that while there was a trend to reduced boost utilization in patients with OPM in comparison to non-OPM patients, overall rates were still high (94.2% vs. 91%; p=0.06) [8]. Furthermore, there were no significant differences in IBTR between OPM and non-OPM patients, supporting similar oncologic outcomes with the adoption of OPM.

The biggest challenge faced in investigating the oncologic impact of OPM with BRT are the low incidence rates of IBTR. In the Cochrane meta-analysis of BRT, an absolute risk reduction (ARR) of 2.5% was found in IBTR incidence rates among all-comers [4]. If one assumes that “perfectly” localized radiotherapy confers an ARR of 2.5%, the assumption would be that any degradation in localization based on OPM could reduce this number, but it is unclear as to what extent. Would there be no benefit or could a lesser benefit still exist? The combination of generally high control rates and potentially small differences in ARR conferred by BRT would mean that definitive results produced by clinical trials or patient data analyses would require very high patient numbers. As such, it seems unlikely that the field can expect a definitive answer in this regard. The results of our study demonstrate that the OPM procedure itself did not impact our radiation oncologists’ decision to administer BRT or the BRT volume relative to the tumor volume. Unfortunately, our data analysis cannot answer the question as to how effective the boost delivery was in target localization or ultimately reduction in IBTR incidence rates.

Without definitive quantification of treatment effect, oncologists have instead relied on first principles, and the primary tenant of radiotherapy, that is, if one intends to treat, one must accurately target the region at risk. The concern with OPM and BRT relates to the architectural distortion in local breast tissue and the implications this has on tumor (and tumor cavity) localization. Several studies have investigated fiducial placement within the tumor cavity at the time of OPM in order to aid in boost delineation at the time of radiotherapy, and results have shown that clip location can be outside the original tumor quadrant in up to 50% of cases, presumably as a result of the rotations and closures that are inherent in this technique [16,17]. Several other localization devices, such as radio-opaque gels and films, have also been investigated as intra-operative markers to aid in future radiation boost planning [19,20]. Consequently, there have been calls for increased collaboration and a multi-disciplinary approach to patient selection for treatment planning in OPM cases [10,21].

Limitations of this study include the observational nature, particularly in regard to BRT indication as there were no firm institutional care pathways at the time of analysis. However, this limitation was mitigated by the strong concordance shown between our retroactively applied “BRT indicated” criteria and patients who actually received BRT. The lack of long-term follow-up in this study is also a limitation, particularly in regard to secondary outcomes of IBTR incidence rates, but as mentioned earlier, with the low anticipated rates of recurrence and expected differences with BRT treatment, it is unlikely that we can expect significant differences with longer follow-up given the number of patients analyzed.

## Conclusions

The decision to treat patients with BRT radiotherapy in addition to standard whole breast irradiation does not seem to be affected by the decision to pursue an oncoplastic procedure as compared to standard BCS. There were no additional negative sequelae such as impact on time to radiation treatment and local recurrence found in this study, although there were few events in the short follow-up reported. Instituting more dedicated tumor cavity localization at the time of surgery may improve accuracy and potentially efficacy, and specific investigation through prospective clinical study presents a unique research opportunity. In the meantime, a multi-disciplinary approach in this patient population is recommended.

## References

[1] (2021). Canadian Cancer Statistics 2018. https://www.cancercareontario.ca/sites/ccocancercare/files/assets/OCS2018_rev13122018.pdf.

[2] Fisher B, Anderson S, Bryant J (2002). Twenty-year follow-up of a randomized trial comparing total mastectomy, lumpectomy, and lumpectomy plus irradiation for the treatment of invasive breast cancer. N Engl J Med.

[3] Bartelink H, Maingon P, Poortmans P. (2015). Whole-breast irradiation with or without a boost for patients treated with breast-conserving surgery for early breast cancer: 20-year follow-up of a randomised phase 3 trial. Lancet Oncol.

[4] Kindts I, Laenen A, Depuydt T, Weltens C (2017). Tumour bed boost radiotherapy for women after breast-conserving surgery. Cochrane Database Syst Rev.

[5] Aristokleous I, Saddiq M (2019). Quality of life after oncoplastic breast-conserving surgery: a systematic review. ANZ J Surg.

[6] Bogusevicius A, Cepuliene D, Sepetauskiene E (2014). The integrated evaluation of the results of oncoplastic surgery for locally advanced breast cancer. Breast J.

[7] Arnaout A, Ross D, Khayat E (2019). Position statement on defining and standardizing an oncoplastic approach to breast-conserving surgery in Canada. Curr Oncol.

[8] Borm KJ, Schönknecht C, Nestler A (2019). Outcomes of immediate oncoplastic surgery and adjuvant radiotherapy in breast cancer patients. BMC Cancer.

[9] Yoon JJ, Green WR, Kim S, Kearney T, Haffty BG, Eladoumikdachi F, Goyal S (2016). Oncoplastic breast surgery in the setting of breast-conserving therapy: a systematic review. Adv Radiat Oncol.

[10] Shah C, Al-Hilli Z, Schwarz G (2018). Oncoplastic surgery in breast cancer: don't forget the boost!. Ann Surg Oncol.

[11] Maguire PD, Adams A, Nichols MA (2015). Oncoplastic surgery and radiation therapy for breast conservation: early outcomes. Am J Clin Oncol.

[12] Schaverien MV, Stallard S, Dodwell D, Doughty JC (2013). Use of boost radiotherapy in oncoplastic breast-conserving surgery - a systematic review. Eur J Surg Oncol.

[13] Veiga DF, Veiga-Filho J, Ribeiro LM (2010). Quality-of-life and self-esteem outcomes after oncoplastic breast-conserving surgery. Plast Reconstr Surg.

[14] Furet E, Peurien D, Fournier-Bidoz N (2014). Plastic surgery for breast conservation therapy: how to define the volume of the tumor bed for the boost?. Eur J Surg Oncol.

[15] Pezner RD (2011). The oncoplastic breast surgery challenge to the local radiation boost. Int J Radiat Oncol Biol Phys.

[16] Pezner RD, Tan MC, Clancy SL, Chen YJ, Joseph T, Vora NL (2013). Radiation therapy for breast cancer patients who undergo oncoplastic surgery: localization of the tumor bed for the local boost. Am J Clin Oncol.

[17] Eaton BR, Losken A, Okwan-Duodu D (2014). Local recurrence patterns in breast cancer patients treated with oncoplastic reduction mammaplasty and radiotherapy. Ann Surg Oncol.

[18] Kapadia SM, Reitz A, Hart A (2019). Time to radiation after oncoplastic reduction. Ann Plast Surg.

[19] Riina MD, Rashad R, Cohen S (2020). The effectiveness of intraoperative clip placement in improving radiation therapy boost targeting after oncoplastic surgery. Pract Radiat Oncol.

[20] Struik GM, Hoekstra N, Klem TM (2019). Injection of radiopaque hydrogel at time of lumpectomy improves the target definition for adjuvant radiotherapy. Radiother Oncol.

[21] Strach MC, Prasanna T, Kirova YM (2019). Optimise not compromise: the importance of a multidisciplinary breast cancer patient pathway in the era of oncoplastic and reconstructive surgery. Crit Rev Oncol Hematol.

